# Advances in protein subunit vaccines against tuberculosis

**DOI:** 10.3389/fimmu.2023.1238586

**Published:** 2023-08-15

**Authors:** Ying Zhang, Jin-chuan Xu, Zhi-dong Hu, Xiao-yong Fan

**Affiliations:** ^1^ Shanghai Public Health Clinical Center, Shanghai Institute of Infectious Disease and Biosecurity, Fudan University, Shanghai, China; ^2^ TB Center, Shanghai Emerging and Re-emerging Infectious Disease Institute, Fudan University, Shanghai, China

**Keywords:** tuberculosis, protein subunit vaccines, antigen epitopes, adjuvants, clinical trials, animal models

## Abstract

Tuberculosis (TB), also known as the “White Plague”, is caused by *Mycobacterium tuberculosis* (*Mtb*). Before the COVID-19 epidemic, TB had the highest mortality rate of any single infectious disease. Vaccination is considered one of the most effective strategies for controlling TB. Despite the limitations of the Bacille Calmette-Guérin (BCG) vaccine in terms of protection against TB among adults, it is currently the only licensed TB vaccine. Recently, with the evolution of bioinformatics and structural biology techniques to screen and optimize protective antigens of *Mtb*, the tremendous potential of protein subunit vaccines is being exploited. Multistage subunit vaccines obtained by fusing immunodominant antigens from different stages of TB infection are being used both to prevent and to treat TB. Additionally, the development of novel adjuvants is compensating for weaknesses of immunogenicity, which is conducive to the flourishing of subunit vaccines. With advances in the development of animal models, preclinical vaccine protection assessments are becoming increasingly accurate. This review summarizes progress in the research of protein subunit TB vaccines during the past decades to facilitate the further optimization of protein subunit vaccines that may eradicate TB.

## 1 Introduction

Tuberculosis (TB), caused by *Mycobacterium tuberculosis* (*Mtb*), has afflicted humans for thousands of years. *Mtb* is highly contagious and colonizes the respiratory tract through airborne droplets (1). TB remains a serious threat to public health, with 10.6 million new cases and 1.6 million deaths reported worldwide in 2021 (2). In addition, enormous challenges for TB prevention and treatment are posed by the emergence of multidrug-resistant TB (MDR-TB) (3), the lack of effective methods for the differential diagnosis of latent TB infection (LTBI) (4), immune disorder caused by co-infection with HIV (5).

The development of the Bacille Calmette- Guérin (BCG) vaccine was a major milestone in the history of global TB control. Even though it has been more than 100 years since BCG was developed, BCG is still the only licensed TB vaccine worldwide. However, its protective efficiency is still controversial due to its limited immune protection in adults (6). Therefore, a more effective TB vaccine that protects against different stages of the disease’s development is urgently needed.

The main TB vaccine candidates include live attenuated vaccines, inactivated vaccines, recombinant viral vector vaccines, and protein subunit vaccines. Since both whole-cell-based and virus-based vaccines pose potential risks to human health, protein-subunit vaccines consisting of protective antigens may be safer and more attractive (7). However, the biggest concern with protein subunit vaccines is inadequate immunogenicity, therefore, optimizing the vaccine composition to trigger a potent and long-lasting immune response is crucial. Novel vaccine adjuvants are powerful tools that may overcome the immunogenicity limitations of protein subunit vaccines.

In this review, we focus on the advances in antigen optimization, adjuvant selection, clinical trials, animal models, and vaccination strategies of protein subunit vaccines, which may foretell the future of TB vaccine research and development.

## 2 Protein epitope optimization strategy

The complex genetic composition, multiple immune evasion strategies, and the lack of rigorous immune markers make the identification of key protective epitopes against *Mtb* a major challenge. Several methods have been used to predict the optimal epitopes for vaccine design.

Although CD4^+^ T cells are necessary to protect against TB, they may not be sufficient to obtain a completely protective immune response (8). Many researchers have focused on identifying antigens that stimulate the CD8^+^ T-cell-mediated responses that also play a protective role against TB and latent TB infection (LTBI) (9). Additionally, a growing number of studies have shown that antibodies produced by B cells contribute to the fight against TB (10). Therefore, vaccines that induce combined CD4^+^, CD8^+^ T-, and B-cell immune responses may be the most effective. Bioinformatics tools enable the rapid analysis of the entire genome and proteome of pathogens to predict potentially protective T- or B-cell epitopes and the character of their specific binding to major histocompatibility complex (MHC) molecules (11).

The structure of an antigen determines the specificity, affinity, and accessibility of the binding sites to MHC or antibody, which affects the potency of the immune response (12). Therefore, antigen geometry can be another critical factor in vaccine design (13). “Reverse vaccinology” was proposed 20 years ago, based on the availability of genome sequence information to design vaccines. With the development and application of immunology, proteomics, systems biology, and structural biology, we have entered the era of “Reverse vaccinology 2.0”, in which the structural features of antigens and antibodies are used to guide the design of recombinant vaccine antigens. Developments in X-ray crystallography, electron microscopy, and computational biology have all contributed (14). Currently, AlphaFold2 is the most advanced protein 3D structure prediction tool (15). By predicting and analyzing the higher configuration of the 3D antigen structure, the linear epitopes for T-cell receptors and the conformational epitopes for B-cell receptors can be comprehensively optimized to improve vaccine protective efficiency (16).

Combining bioinformatics, structural information, and the AlphaFold2 prediction model to obtain the structural basis underlying protective immune responses to key epitopes is now a popular design strategy to get efficient, long-term, and broad-spectrum responses with multi-epitope TB protein subunit vaccine candidates (14–17).

## 3 Protective antigens of *Mtb*

The composition of *Mtb* is complex, and many components exhibit immunogenicity. According to different characteristics and associated growth states, *Mtb* antigens are mainly divided into the following types:

### 3.1 Antigens on the cell wall and capsule

The cell wall and capsule of *Mtb* contain a large number of glycolipids, lipoproteins, and glycoproteins such as cord factor, phthiocerol dimycocerosates, phosphatidylethanolamine, diacyl trehaloses, lipoarabinomannan, phosphatidyl-myoinositol mannosides, and heparin-binding adhesin, etc. (18, 19) They can activate immune responses and serve as candidate antigens or adjuvants for TB vaccines.

### 3.2 Secretory antigens

*Mtb* can secrete numerous proteins, some of which can inhibit or induce the host immune response by promoting immune escape or activating immune signaling pathways, respectively. Most of the candidate proteins for existing TB vaccines are based on those found as secreted antigens during logarithmic growth of *Mtb*, such as Ag85A/B, ESAT-6, CFP10, TB10.4, MPT64, and PPE18 (20). The secretory antigens are ideal candidate antigens for the recombinant protein subunit vaccine because of their strong immunogenicity and ease of heterologous expression and amplification.

### 3.3 Dormancy phase antigens

The antigens modulated under the DosR regulon are the main proteins involved in the dormant survival process of *Mtb*. A total of 48 structural proteins are known to be involved in aerobic respiration and carbon monoxide inhibition; representative genes include *HspX*, *Rv2623*, *Rv2660c*, etc. (21) Members of the durable hypoxia response (EHR) regulon are structural genes induced after exposure to hypoxia. EHR proteins are presumed to be involved in the adaptation and survival of bacteria during a long-term bacteriostatic process (22). Members of the DosR and EHR regulons are considered promising antigens to be incorporated into protein subunit vaccines for treating LTBI (23).

### 3.4 Resuscitation phase antigens

Resuscitation promotion factors (Rpfs) are involved in the resuscitation and reactivation of dormant *Mtb* infection and induce specific humoral and cellular immune responses in individuals with LTBI (24). There are 5 Rpf-like proteins (RpfA, RpfB, RpfC, RpfD, and RpfE) with partially overlapping functions in *Mtb*. Rpfs, especially RpfB, can trigger a memory T-cell response and has been hypothesized to be an essential antigenic target controlling bacterial activation. Rpfs can be used as candidate antigens for protein subunit vaccines against LTBI infection (25).

### 3.5 BCG regions of difference (RD) antigens

BCG strains have structures similar to *Mtb*, but 16 genomic region of difference (RD) antigens are deficient in BCG compared to *Mtb* (26). The RD1 gene products contain a variety of potential virulence factors, such as ESAT-6 and CFP10 (26). They play multiple roles in *Mtb* progression and pathogenicity, and are considered suitable candidates for use in treatment and diagnosis (27). The poor protective effect of BCG may be related to the loss of a large number of genes encoding protective antigens. Therefore, RD antigens should be emphasized in constructing recombinant protein subunit vaccines.

## 4 Adjuvants

With the limitations in immunogenicity and bioavailability, excellent adjuvants are critical for protein subunit vaccines. Alum has been the only licensed adjuvant in human vaccines for several decades. However, it has been considered unsuitable for vaccines against intracellular pathogens such as *Mtb* due to its insufficient ability to induce Th1 cellular immunity and CD8^+^ cytotoxic responses. TB-specific adjuvants that induce a strong immune response in the lungs but minimize the corresponding tissue damage are ideal. In order to meet the needs of TB vaccine development, a workshop entitled “Vaccine Adjuvants for Advancing the treatment of Mycobacterium tuberculosis” was held in July 2020, and factors correlates of protective immunity, targeting specific immune cells, immune evasion mechanisms, and animal models were identified as four research areas critical to the development of optimal TB vaccine adjuvants (28). In recent years, a variety of novel adjuvants have been developed, and most available protein subunit vaccine adjuvants are based on Toll-like receptor (TLR) agonists and use liposomes and emulsions as delivery vehicles as shown in **Table 1**. In addition, nanoparticle-based adjuvants have received extensive attention in recent years, and various novel nanoadjuvants have been used in some of these vaccines.

**Table 1:** Protein subunit vaccine candidates undergoing pre-clinical and clinical trials.

Vaccine Candidates	Antigens	Adjuvants	Adjuvant components	Adjuvant targets	Immune responses	Immunization strategies	Trial phases	References
CysVac2/Advax	CysD, Ag85B	Advax	Delta isoform of inulin formed cationic particles (1-2 μm)	–	IL-17-secreting lung-resident CD4^+^ memory T cells (IFN-γ, TNF-α, IL-2, IL-17)	Prevention, and therapeutic	Pre-clinical	(29–35)
LT70	ESAT-6, Ag85B, peptide 190–198 of MPT64, Mtb8.4, Rv2626c	DDA/PolyI:C	DDA and PolyI:C	TLR-3	CD4^+^ T cells (IFN-γ, IL-2) and antigen-specific IgG1 and IgG2c	BCG booster Therapeutic	Pre-clinical	(36–41)
CFMO-DMT	Rv2875, Rv3044, Rv2073c, Rv0577	DMT	DDA, MPL and TDB	TLR-4	CD4^+^ T cells (IFN-γ, IL-2, TNF-α, IL-17A) and IFN-γ^+^ CD8^+^ T cells	Prevention, therapeutic, and prevent recurrence	Pre-clinical	(42–45)
H64/H74:CAF01	H64 (EsxA, EspD, EspC, EspE, EspR, PE35); H74 (EspB, EsxA, EspD, EspC, EspA, EspR)	CAF01	DDA and TDB	Mincle	CD4^+^ T cells (TNF-α, IL-2)	BCG booster	Pre-clinical	(46–49)
H107	PPE68, ESAT-6, EspI, EspC, EspA, MPT64, MPT70, MPT83	–	–	–	Less-differentiated CD4 Th1 cells and increased Th17 responses	BCG booster	Pre-clinical	(50)
AEC/BC02	Ag85B, ESAT-6-CFP10	BC02	CpG DNA fragment and aluminum salt	TLR-9	CD4^+^ T cells (IFN-γ, IL-2) and antigen-specific IgG, IgG1, and IgG2a	Prevention, and therapeutic	Phase I	(51–55)
ID93+GLA-SE	Rv2608, Rv3619, Rv3620, Rv1813	GLA-SE	GLA in a stable oil-in-water SE	TLR-4	CD4^+^ T cells (IFN-γ, TNF-α, IL-2) and antigen-specific IgG1 and IgG3	BCG booster, therapeutic, and prevent recurrence	Phase IIa	(56–63)
H56:IC31	Ag85B, ESAT6, Rv2660c	IC31	Antimicrobial peptide KLKL5KLK and ODN1a	TLR-9	CD4^+^ T cells (IFN-γ, TNF-α, IL-2) and antigen-specific IgG	BCG booster, therapeutic, and prevent recurrence	Phase IIb	(64–72)
M72/AS01_E_	Mtb 32A, Mtb 39A	AS01E	MPL and the saponin component QS21 co-prepared in cholesterol	TLR-4	CD4^+^ T cells (IFN-γ, IL-2, TNF-α, IL-17), CD8^+^ T cells (IFN-γ or TNF-α), NK cell IFN-γ, and antigen-specific IgG	BCG booster, and therapeutic	Phase IIb	(73–82)
Gam TBvac	ESAT-6, CFP10, Ag85A	Dextran/CpG	DEAE-dextran polymer associating with BCG-derived unmethylated CpG oligodeoxynucleotide	TLR-9	CD4^+^ T cells (IFN-γ, IL-2, TNF-α), CD8^+^ T cell IFN-γ, and antigen-specific IgG	BCG booster	Phase IIb	(29, 83–87)

DDA, dimethyldioctadecylammonium; PolyI:C, polyinosinic-polycytidylic acid; MPL, ligand3-O-desacyl-4′-monophosphoryl lipid A; TDB, trehalose-6,6-dibehenate; CpG, cellular guanine phosphate; GLA, Glucopyranosyl Lipid A; SE, squalene emulsion; ODN1a, oligodeoxynucleotide (ODN) 1a; DEAE, Polycationic diethylaminoethyl.

### 4.1 CAF01

CAF01 comprises cationic surfactant lipid-based liposomes dimethyldioctadecylammonium (DDA) and glycolipid immunomodulator trehalose-6,6-dibehenate (TDB). DDA is a potent adjuvant capable of eliciting cellular and humoral immune responses (46). TDB is a synthetic analog of mycobacterial cord factor that is located in the cell wall of mycobacteria and has intrinsic immunostimulatory properties that activate Mincle (47). TDB incorporated with DDA creates a stable liposome by forming hydrogen bonds between the liposome membrane and the surrounding water. CAF01 has been shown to generate a Th1/Th17 polarization response via Mincle-dependent IL-1 production and subsequent MyD88 signaling (48).

### 4.2 AS01 and DMT

AS01 is a liposome-based adjuvant that consists of the 3-O-desacyl-4′-monophosphoryl lipid A (MPL) and the saponin QS-21 (*Quillaja saponaria* extract), co-prepared in the presence of cholesterol (73). MPL acts as a TLR4 agonist, stimulates NF-κB transcriptional activity, and induces a Th1 response. QS-21 can enhance the antigen presentation ability of antigen-presenting cells (APCs) and activate/differentiate T cells towards Th1 immune responses. DMT is a combination of the MPL, DDA, and TDB that provides more potent and longer-lasting protective efficacy, including antigen-specific CD4^+^ Th1 response, IFN-γ^+^ CD8^+^ CTL response, and limited humoral response (42).

### 4.3 GLA-SE

GLA-SE is a mixture of the TLR4 agonist glucopyranosyl lipid A (GLA) and squalene emulsion (SE) (56). GLA is a synthetic lipopolysaccharide (LPS) derivative that maintains vigorous immunostimulatory activity and has low toxicity (57). SE is able to increase the secretion of proinflammatory cytokines such as IL-6, IL-12, and TNF (58). Both GLA and SE alone can promote IgG2 response, while the combination of GLA-SE can induce considerable Th1 response (56).

### 4.4 IC31

IC31 comprises the synthetic, positively charged antimicrobial peptide KLKL5KLK and oligodeoxynucleotide 1a (ODN1a) (64). ODN1a is an immune stimulatory molecule that promotes Th1-biased immune responses through the TLR9/MyD88 pathway. KLK can act as an immune stimulator that aids transfer into cells in the absence of cell membrane permeability, allowing more efficient functioning of intracellular TLRs (65). KLK induces a Th2 immune response when used alone and a stronger Th1 and Th2 immunity when combined with ODNla (66, 67).

### 4.5 Dextran/CpG

Dextran/CpG is a novel adjuvant developed with diethyl aminoethyl (DEAE)-dextran and CpG ODN (83). CpG is a TLR9 agonist with the ability to promote Th1 immune responses (secretion of IFN-γ, TNF-α, and IL-12 cytokines), opsonizing antibodies (IgG2a), and potent CD8^+^ T cell responses (84). Dextran interacts with DC-SIGN family receptors, mannose receptors, and langerin, all triggering innate immunity that promotes inflammation. Furthermore, Dextran/CpG adjuvant enhances activation of lymph node-resident APCs, thus enhancing T-cell priming (29, 83).

### 4.6 Advax

Advax is a novel cationic adjuvant based on the Delta inulin isoform and has a diameter of about 1-2 μm (30). Advax-based adjuvants have been shown to promote protective immunity against several pathogens in various animal species (31, 32). The potent chemotactic effect induced by Advax enables leukocyte recruitment to the site of inoculation and elicits a broad range of immune responses, including humoral response, Th1, Th2, and Th17 T-cell responses (31).

### 4.7 BC02

BC02 consists of BCG-derived unmethylated CpG DNA fragments and aluminum salts (Al(OH)_3_) (51). CpG tends to induce Th1-type immune responses, while alum skews the response to promote the Th2 response to secrete IL-4 and IL-5 cytokines and produce IgG1 and IgE-type antibodies (52). BC02 induces robust Th1 and Th2 responses with acceptable safety (51).

### 4.8 DDA/poly(I:C)

DDA/poly(I:C) is composed of cationic liposome vector DDA and polyriboinosinic acid–polyribocytidylic acid, poly(I:C). Poly(I:C) mimics viral dsRNA and is a promising immune stimulator candidate for vaccines against intracellular pathogens. Poly(I:C) signaling primarily depends on TLR3 and melanoma differentiation-associated gene-5 (MDA-5) (36). Moreover, poly(I:C) induces strong Th1-skewed immune responses, with enhanced IFN-γ, IL-6, IL-12p70 as well as high antigen-specific IgG antibody (37, 38).

### 4.9 Nanoadjuvants

With the development of nanotechnology and the increasing understanding of immune responses to metals, different types of inorganic nanoadjuvants have been developed, including manganese (88), iron (89), silicon (90), magnesium (91), and gold-based adjuvants (92), etc. The commonly used polymers are poly-lactic-co-glycolic acid (PLGA), which can be constructed into nano- or larger particles to improve immune response efficiency (93). Compared with the traditional adjuvants, the novel inorganic nanoadjuvants can better activate both humoral and cellular immunity, induce a more balanced Th1/Th2 immune response and improve the safety and effectiveness of vaccines (94). Inorganic nanoadjuvants have been used in vaccines for various diseases, such as coronavirus (95), cancer (91, 96), and pertussis (97). Nanoadjuvants for TB vaccines are also being developed to enhance the immune response and extend the duration of protection (98, 99).

## 5 Pre-clinical and clinical trials

Pre-clinical and clinical trials are always needed to evaluate the safety and efficacy of novel vaccine candidates. We summarize the significant progression of protein subunit vaccines in recent trials.

### 5.1 Pre-clinical phases

#### 5.1.1 CysVac2/Advax

CysD is an important protein in the sulfur assimilation pathway of *Mtb* that is up-regulated during LTBI (33). CysVac2, which consists of CysD and the acute phase antigen Ag85B, is an effective prophylactic and therapeutic vaccine, particularly effective in controlling an advanced infection (34). Notably, administration of CysVac2 to mice previously infected with TB significantly reduced bacterial load and immunopathological damage in the lungs compared to mice vaccinated with BCG (33). CysVac2 with Advax elicited multifunctional CD4^+^ T cells with enhanced secretion of IFN-γ, TNF, and IL-2. Moreover, CysVac2/Advax induced the accumulation of lung-resident memory T cells expressing IL-17 and RORγT before and after the *Mtb* aerosol challenge (35). Thus, CysVac2/Advax was shown to be a suitable vaccine candidate for the control of TB pulmonary infection.

#### 5.1.2 LT70

LT70 is a multistage protein subunit vaccine composed of antigens prominent at different metabolic stages of the *Mtb* life cycle, including ESAT-6, Ag85B, peptide 190-198 of MPT64, proliferative phase antigen Mtb8.4 and latency-associated antigen Rv2626c, with DDA/Poly(I:C) as an adjuvant (39). In a murine model, LT70 induced robust antigen-specific humoral (secretion of IgG1 and IgG2c antibodies) and Th1 cell immunity response (IFN-γ, IL-2) with immune protection against *Mtb* infection superior to that provided by BCG. When used as a booster vaccine, it enhanced the protective effect of BCG by reducing the bacterial load in the lungs of mice (39). Another study showed that LT70 had a significant therapeutic effect on LTBI in mice (40). In addition, prolonged LT70 inoculation intervals (0-4-12w) produced stronger protective effects and tended to induce long-term central memory T cells (T_CM_, stronger IL-2 secretion capacity) rather than effector memory T cells (T_EM_, stronger IFN-γ secretion capacity) (41).

#### 5.1.3 CFMO-DMT

CMFO is a multistage subunit vaccine (containing Rv2875, Rv3044, Rv2073c, and Rv0577) administered subcutaneously adjuvanted with DMT (43, 44). CMFO-DMT could induce the immune response of IFN-γ^+^ or IL-2^+^ CD4^+^ T cells and IFN-γ^+^ CD8^+^ T_EM_ cells in spleen more effectively than BCG (43–45). CMFO-DMT prevented *Mtb* reactivation by eliminating the bacterial load from the lung and spleen in LTBI mice (43), suggesting CMFO-DMT is a promising adult TB vaccine candidate for preventive and therapeutic purposes.

#### 5.1.4 H64/H74/H107

H64 (EsxA, EspD, EspC, EspE, EspR, and PE35), H74 (EspB, EsxA, EspD, EspC, EspA, and EspR), and H107 (PPE68, ESAT-6, EspI, EspC, EspA, MPT64, MPT70, and MPT83) are protein subunit vaccines composed of *Mtb*–specific antigens (49, 50). H64, and H74 showed comparable protection to H65 (consisting of antigens also present in BCG) in mice and guinea pigs. However, when used as a BCG booster vaccine, H65-induced highly differentiated CD4^+^ T cells that did not contribute to the protective effect of BCG, while H64 and H74 induced less differentiated and versatile CD4^+^ T cells (secreting TNF-α alone or TNF-α and IL-2 in combination) with a protective effect against *Mtb* pulmonary infection (49). H107 vaccination also significantly increased the clonal diversity of the BCG-induced CD4^+^ T cell repertoire, including Th17-responsive and poorly differentiated memory CD4^+^ Th1 cells (50). Therefore, protein subunit vaccines containing *Mtb*-specific antigens may have more potential to serve as booster vaccines in BCG-primed populations.

### 5.2 Phase I clinical trials

#### 5.2.1 AEC/BC02

AEC/BC02 is a vaccine candidate for LTBI consisting of Ag85B and the fusion protein ESAT-6-CFP10 with adjuvant BC02 (53). Preclinical studies have shown that AEC/BC02 can induce long-term antigen-specific cellular immune responses in mice. In addition, AEC/BC02 reduced the risk of the Koch phenomenon in a guinea pig LTBI model (51, 54). In a murine LTBI model, after AEC/BC02 therapy, the bacterial load in the spleen and lung was significantly reduced. Furthermore, AEC/BC02 induced a significant Th1 response with antigen-specific release of IFN-γ, IL-2, and IgG (IgG1, and IgG2) (55). A phase Ib clinical trial evaluating the safety and immunogenicity of AEC/BC02 in healthy adults has been completed (NCT04239313), and volunteers are currently being recruited for phase II trials.

### 5.3 Phase II clinical trials

#### 5.3.1 ID93+GLA-SE

ID93+GLA-SE comprises four *Mtb* antigens (Rv2608, Rv3619, Rv3620, and Rv1813) with GLA-SE as an adjuvant (59). In mice and guinea pigs, ID93+GLA-SE protected against *Mtb* virulent strain H37Rv and multidrug-resistant strain TN5904 (60). ID93+GLA-SE combined with the first-line anti-TB drugs rifampicin and isoniazid showed therapeutic efficacy in *Mtb*-infected mice and nonhuman primate (NHP) models (61). ID93+GLA-SE was found to provide long-lasting protection by inducing antigen-specific IgG1 and IgG3 and multifunctional CD4^+^ T cell responses with enhanced IFN-γ, TNF, and IL-2 secretion in a phase I trial (59, 62). A phase IIa trial showed that ID93+GLA-SE enhanced therapeutic efficacy and reduced disease recurrence by inducing robust cellular and humoral immune responses (63). Phase IIb trials that are aimed at preventing TB recurrence are currently in preparation.

#### 5.3.2 H56:IC31

H56:IC31 is formed by Ag85B, ESAT-6, and Rv2660c protein fusion with adjuvant IC31. Due to the presence of the latency-associated protein Rv2660c, a protective effect of H56 in the murine LTBI model was expected and this was observed (68). In NHP aerosol challenge models, H56:1C31 limited the development of advanced infection and LTBI (69). In a phase I trial, the vaccine induced antigen-specific IgG and CD4^+^ T cell responses (IFN-γ, TNF-α, IL-2) (70). In a phase I/IIa clinical trial, variations in the dose and time of H56:IC31 inoculation were studied. Two to three vaccination doses were optimal with acceptable safety and tolerability (71). Phase IIb trials of H56:IC31 to reduce TB recurrence in HIV-negative patients receiving anti-TB chemotherapy are ongoing (NCT03512249) (72).

#### 5.3.3 M72/AS01_E_

M72/AS01_E_ is composed of two immunogenic *Mtb* fusion proteins (Mtb32A and Mtb39A) with AS01_E_ as an adjuvant. M72/AS01_E_ protected against *Mtb* invasion after aerosol infection when administered intramuscularly to C57BL/6 mice and guinea pigs (74). When used as a BCG booster vaccine, M72/AS01_E_ provided long-term protection and improved guinea pig and NHP survival post *Mtb* infection (75, 76). The vaccine was protective against TB in adults in a phase II trial, but the trial was suspended because local reactions were observed in some vaccinated individuals (77). The safety and immunogenicity of M72/AS01_E_ were evaluated in HIV-negative adolescents in TB-endemic areas. The results showed that M72/AS01_E_ was safe and could induce M72/AS01_E_ -specific IgG antibody, CD4^+^ (IFN-γ, TNF-α, IL-2 and/or IL-17), CD8^+^ (IFN-γ, TNF-α) T-cells and antigen-dependent NK cell IFN-γ production (78). Another phase II trial, in India, showed elevated cellular and humoral responses by M72/AS01_E_ in both HIV-negative and HIV-positive individuals that persisted for 3 years with no safety concerns (79). Subsequently, a Phase II clinical trial showed that M72/AS01_E_ provided 54% protection against progression to active pulmonary TB in LTBI adults, without significant adverse effects (80). In a randomized placebo-controlled phase IIb study, M72/AS01_E_ protected adults against active TB by 49.7% for at least 3 years without serious safety concerns (81). However, it is doubtful that the excellent protection of M72/AS01_E_ is mainly based on data from a single population, and large-scale long-term trials in a wider population are needed (82).

#### 5.3.4 GamTBvac

The GamTBvac vaccine combines TB antigens ESAT-6, CFP10, and Ag85A with a novel adjuvant, dextran/CpG. GamTBvac showed significant immunogenicity and protection in *Mtb*-infected mice and guinea pigs when used as a BCG booster vaccine (85). GamTBvac was found to be immunogenic and safe in a phase I trial in BCG-vaccinated, uninfected healthy people (86). A completed phase IIa trial showed that GamTBvac was safe and had considerable immunogenicity in inducing CD4^+^ T cells expressing Th1 cytokines (IFN-γ, IL-2, and TNF-α), CD8^+^ T cells secreting IFN-γ, and IgG responses (87). Phase III trials to evaluate the vaccine’s protective efficacy against TB in large populations are currently enrolling volunteers (NCT04975737).

## 6 Animal models

**Figure 1:**
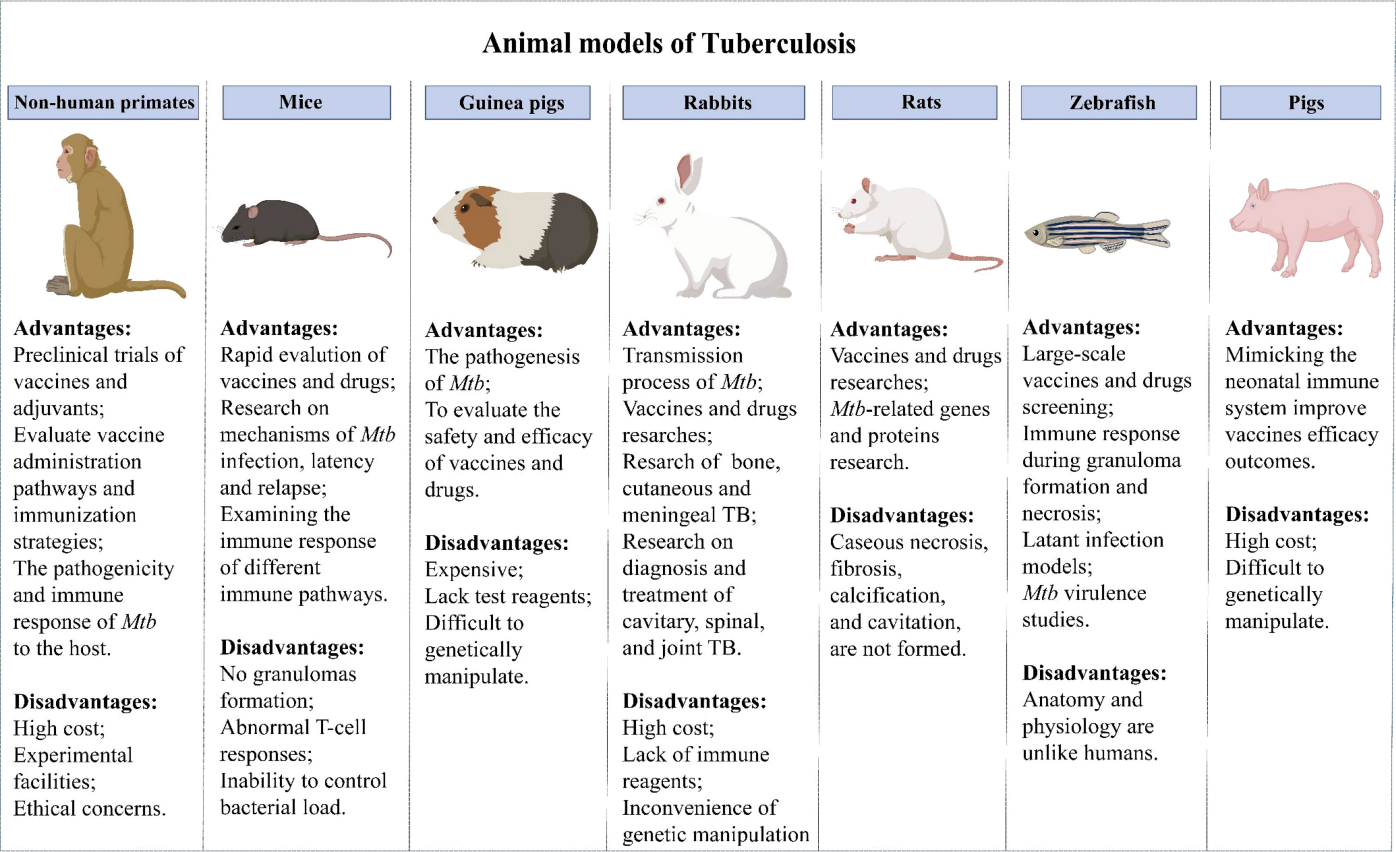
Animal models of Tuberculosis.

Evaluating vaccine safety and protection in animal models is obligatory before a vaccine enters clinical trials. The development of animal models of TB has advanced the understanding of host responses to *Mtb* infection and accelerated the development of TB vaccines. Currently, many animal models are used for TB vaccine evaluation (**Figure 1**).

### 6.1 Mice

Mice provide the most widely used models due to the advantages of relatively low price, short experiment cycle, mature immunological evaluation indicators, abundant commercial reagents and genetically modified inbred strains (100). The mouse strains most commonly used in evaluating the immune efficacy of TB vaccines are BALB/c and C57BL/6, which are sensitive to TB vaccines immunization routes (101). However, the immune response induced by TB vaccine was different in BALB/c and C57BL/6 mice. M Carmen Garcia-Pelayo et al. found that although BCG present equally protective in BALB/c and C57BL/6 mice, it was display more enhanced Th1 and Th17 response in BALB/c mice than C57BL/6 mice (102). In another study, ChAdOx1.PPE15 as a booster vaccine for BCG improved the efficacy of BCG in C57BL/6 mice, but not in BALB/c mice (103). The susceptibility to TB and the protective responses to the vaccines vary according to the route of infection and immunization. Subcutaneous immunization is the most classic immunization method for TB vaccine, but mucosal immunization has received extensive attention in the pathogenic bacteria infected by mucosal route. A multrivalent chimpanzee adenovirus vectored vaccine developed by Sam Afkhami et al. showed strong protection against both replicating and dormant *Mtb* through mucosal immunization (104). Previous research by Claudio Counoupas et al. have shown that intratracheal instillation of CysVac2/Advax protected mice more effectively than the intramuscular vaccine (35).

However, despite a high genetic similarity between mice and humans, significant differences in clinical immune responses between mice and humans have stalled clinical trials of many novel vaccines that had previously shown considerable efficacy in murine models. To overcome this problem, humanized mouse models have been extensively studied in recent years. Humanized mice have a reshaped immune system, making the immune responses more like those of humans. They have been widely used in studies of epitopes and epitope-based TB protein subunit vaccine development (105). Although the use of humanized murine models has enabled many advances in TB vaccine research, deficiencies in the models such as the inability to establish LTBI and granulomas (100), abnormal T-cell responses, and the inability to control bacterial load have limited their use.

### 6.2 NHPs

NHPs can better represent the human immune responses for assessment of the safety and efficacy of TB vaccines and adjuvants due to the close genetic and pathophysiological similarities between NHPs and humans. Rhesus macaques (RM) and cynomolgus macaques (CM) are the most commonly used primate models for TB vaccine research. It is well known that there are differences between macaque species in their ability to control disease progression, with RM showing higher rates of progression and higher levels of bacterial burden compared to CM (106). RM are often used in vaccine evaluation studies because the results of infection are more uniform than CM, while RM are often used in drug evaluation studies because they are better able to control the disease (107). NHPs provide essential insights into host-pathogen interactions during TB infection by simulating the pathogenesis of TB in humans, including the occurrence of LTBI and granuloma formation (108). NHPs can be used to evaluate the immune effect of different vaccine administration pathways and immunization strategies (75, 109).

The use of the NHP models has brought some breakthroughs in TB vaccine development in recent years. First of all, the preclinical evaluation of novel vaccines by the NHP model has facilitated the transformation of vaccines to prevent and therapy *Mtb* infection (110–112). The ultra-low dose aerosol-infected NHP model better simulates the course of human infection with TB and can accurately evaluate the vaccine immune efficacy (113). Moreover, using NHP makes it possible to study the interactions of cells within lung granulomas, which cannot be done in human samples. Laura Hunter et al. used infection in RM and CM models to determine the basic composition of granulomas induced after infection with the *Mtb* Erdman strain, as well as the spatial distribution of immune cells in granulomas in RM and CM and changes over time (114). This informs research into TB vaccines and treatments, and may provide novel immunotherapy strategies against TB. Furthermore, the development of body scanning technology, particularly the combination of PET and CT scans, has made it possible to quantitatively evaluate the protective efficacy of TB vaccines in NHP models (115–117). This strategy allows vaccine evaluation in less time and at a lower cost. However, the high cost of the animals and experimental facilities, as well as the limited quantity available, have hindered their widespread application.

### 6.3 Guinea pigs

Guinea pigs are also a commonly used animal model in the study of TB. Guinea pigs are more susceptible to *Mtb* than mice and can form classical granulomas similar to humans (118). Therefore, they are suited to studies of the pathogenesis of TB and the assessment of vaccines and drugs (119). Guinea pigs have also been used to study the response of Ag-specific T cells to mycobacterium lipids and lipopeptide-rich Ag preparations (120). Diabetes can fuel TB epidemics, and T2D co-infection with TB has been modeled in guinea pigs in recent years and used to test novel therapy approaches (121–123). However, guinea pigs are more expensive, lack test reagents, and are more difficult to genetically manipulate than mice. Adjuvant subunit vaccines tend to be less protective in guinea pigs than in mice, resulting in few successful trials of adjuvants in guinea pig models (60, 124). The cause of the limited protective immunity provided by adjuvants in guinea pig models awaits clarification, and more tools and reagents are needed for guinea pig models.

### 6.4 Pigs

The immunity to *Mtb* infection in neonates is markedly distinct from that in adults. Innate and adaptive immune responses in infants cannot be inferred from adult human or animal models (125). Due to their high similarity to humans in terms of anatomy, genetics, and immune response, pigs are widely used in numerous studies (126, 127). The isolated and sterile state of the porcine fetus during pregnancy is conducive to the study of the interaction between the immature immune system and microorganisms and to determine the changes in the immune structure and function during fetus development (128). Surprisingly, pigs can undergo pathological changes to *Mtb* infection including caseous necrosis, liquefaction, and cavitation and mimic the immune response of vaccination BCG in humans (129). Mimicking the human neonatal immune system in pigs could improve our understanding of the infant immune response to TB. More neonatal and early-life animal models are needed to advance the development of anti-TB vaccines and drugs for neonates.

### 6.5 Other animal models

Other animal models, such as rabbits, rats, and zebrafish, have also been used in *Mtb* vaccine evaluation. Depending on the characteristics of each model, they have been used in different ways. Rabbits are usually infected with *Mtb* by aerosol route (130), and susceptibility to *Mtb* varies among different populations (131). Most rabbits available today are highly resistant to infection with *Mtb* (As Lurie’s - susceptible breed have become extinct), but highly susceptible to infection with the closely related *Mycobacterium bovis*. They can form granulomas, liquefaction, and cavities similar to the events found in humans, making them suitable for the study of processes leading to transmission of *Mtb* as well as for vaccine and drug research. In addition, the rabbit model has been used in studies of cavitary, spinal, joint, cutaneous, and meningeal TB (132, 133). However, due to the high cost, lack of immune reagents, and the inconvenience of genetic manipulation, the utility of the rabbit model is limited.

The types of rats commonly used in TB studies are American cotton rats, Wistar rats and diabetic rat strains. Several studies have found that rats exhibit delayed hypersensitivity to *Mtb* infection (134). *Mtb* infected rats can form well-organized granulomas, including epithelioid cells, multinucleated giant cells and foam macrophages, etc., which provide a common research object for the study of host control of *Mtb* and the establishment of latent infection (135). Rats are suitable for TB-related gene and protein research and have the advantages of low cost and simple blood collection, befitting vaccine and drug research (135). Yet, pathological changes in human lungs, such as caseous necrosis, fibrosis, calcification, and cavitation, are not formed in rats.

Recently, zebrafish have attracted increased attention as an animal model for TB. Zebrafish infected with *M. marinum* can form a typical granulomatous structure, which provides an excellent model for scientists to further study the mechanism of granulomatous formation (136, 137). Moreover, zebrafish have the advantages of visual monitoring, convenient genetic manipulation, fast reproduction, and low cost, they are now widely used for bacterial virulence studies and large-scale vaccine and drug screening. The immune responses during granuloma formation and necrosis can be well monitored, making zebrafish one of the best choices for studying latent TB infection (138, 139). Nevertheless, anatomical and physiological differences between zebrafish and humans impede the application of zebrafish models for vaccine development.

### 6.6 Ultra-low dose infection models

TB is characteristically caused by respiratory infection when the smallest aerosol droplets containing only 1 or 2 colonies reach the alveolar spaces (1). Hence, the high-dose challenge that has been typically used in animal models might have contributed to discrepant results between pre-clinical and clinical trials. To better simulate the natural human infection process, ultra-low dose infection models have been developed. Infection of conventional mice with 1-3 CFU *Mtb* produced granulomas with well-defined boundaries similar to human granulomas (140). In addition, the ultra-low dose aerosol-infected NHP model more closely mimicked the process of human natural TB infection. It is being used as a precise and sensitive system to assess the effectiveness of TB vaccines (113).

## 7 Vaccination strategies of protein subunit vaccines

**Figure 2:**
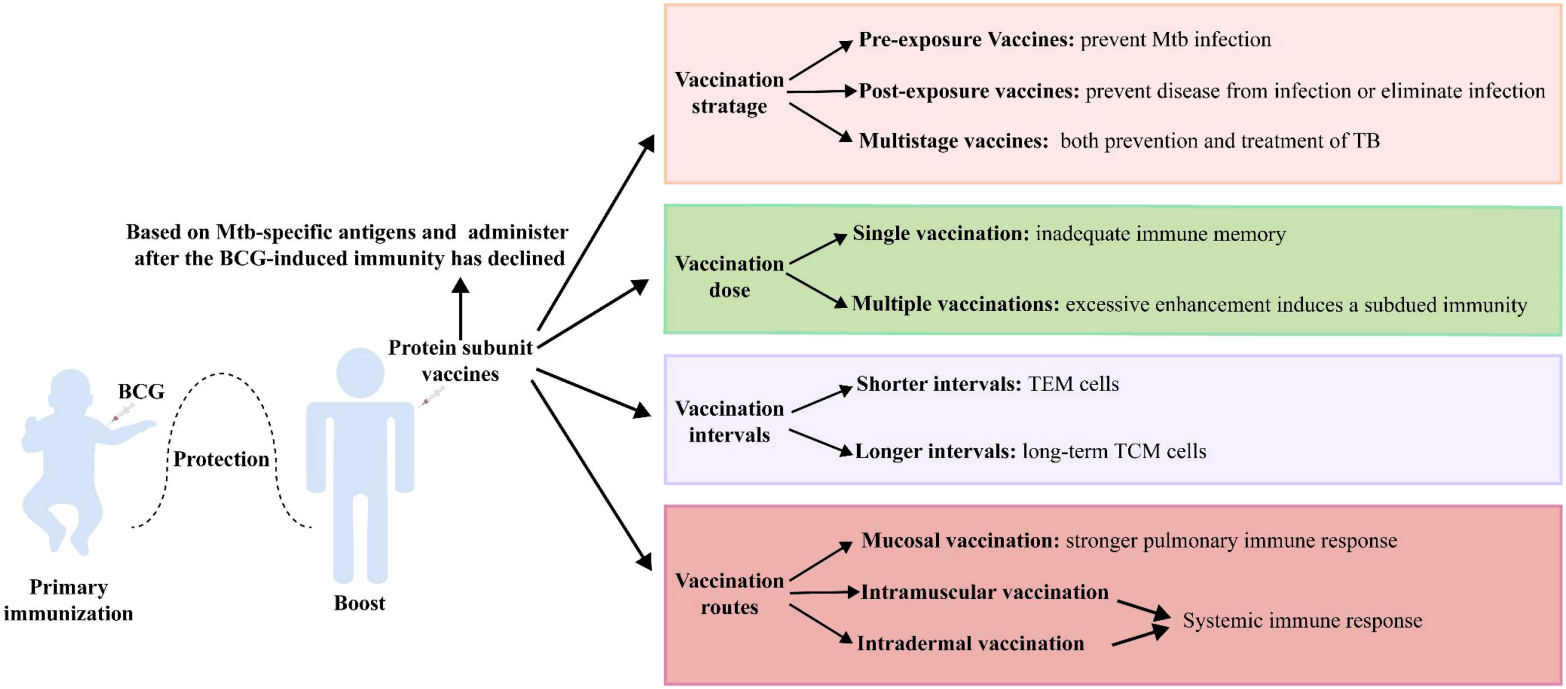
Vaccination strategies of protein subunit vaccines.

Vaccination strategies are critical to the effectiveness of protein subunit vaccines (**Figure 2**). *Mtb* metabolism is profoundly influenced by the different pathophysiological states in different stages of infection. Although current TB vaccines mainly consist of early secreted antigens of *Mtb* and are used for prevention of infection, they are less than ideal in controlling LTBI and active TB. Protein subunit vaccines currently in clinical trials have jumped out of this framework, with M72/AS01E prevents latent infected people exposed to *Mtb* from developing active pulmonary TB disease, and ID93+GLA-SE and H56:IC31 showing promise in the treatment of people with active TB infection. Even more promising is the fact that several multistage protein subunit vaccines comprised of *Mtb* antigens expressed in early growth, dormancy, and resuscitation phases for both prevention and treatment of TB infection have entered pre-clinical (CysVac2, LT70, and CMFO) and clinical trials (H56 and ID93) (35, 39, 43, 63, 71).

Protein subunit vaccines are often used as booster vaccines after BCG priming, and when the antigen in the booster vaccine is shared with BCG, its boosting effect is impaired. BCG vaccination induces highly differentiated CD4^+^ Th1 cells, and the functional plasticity of these cells is limited. Moreover, BCG-generated immunity impedes the subsequent induction of additional protective T cells with memory and lung homing potential by the booster vaccine (141). Therefore, the development of protein subunit vaccine candidates based on *Mtb*-specific antigens (such as H64, H74, and H107) may circumvent this dilemma (49, 50).

Preclinical and clinical trials have shown that some protein subunit vaccines (H56, LT70, CFMO) can elicit more robust protection than BCG when used alone, suggesting that such a vaccine could use as an alternative to BCG (39, 43, 68). However, BCG has definite efficacy against childhood TB and is almost universally given to infants as soon as they are born, so that replacement with an alternative vaccine presents ethical and practical challenges. Consequently, a protein subunit vaccine is more likely to use initially as a booster vaccine. Tests have shown that the protective effect of a BCG-booster vaccine is more pronounced when the immune response to BCG is attenuated (49, 142). One explanation for this could be that reduced levels of BCG-induced immunity open the opportunity for protein subunit vaccines to initiate less differentiated T-cell responses. Therefore, it seems more reasonable to administer the protein subunit vaccine after the BCG-induced immunity has declined (49).

The dose and time of boosting with a protein subunit vaccine are also pivotal factors affecting the effect. Multiple vaccinations are usually required to obtain a substantial immune memory with protein subunit vaccines. However, excessive enhancement induces the production of Tregs, leading to a subdued protective effect of the vaccine (143). Moreover, the interval between vaccinations may impact the type of immunological memory. The strategy with protein subunit vaccines is usually a 2- or 3-week booster regimen, which elicits more T_EM_ cells. A booster regimen with longer intervals of 4 weeks appeared to favor the generation of long-term T_CM_ cells (41).

Finally, different vaccination routes exert a significant influence on efficacy. *Mtb* is transmitted through the respiratory tract, and the protective effect of specific B-cell and strong central memory CD4^+^ and CD8^+^ T-cell responses activated by respiratory mucosal vaccination against *Mtb* infection should be an important consideration (144, 145). Zhang Y et al. found that Ag85A-Mtb32 in adenoviral vectored TB vaccine was more likely to induce systemic immune response through subcutaneous and muscular inoculation, while oral and nasal mucosal immune pathways induced stronger pulmonary immune response (105). Moreover, trained immunity was more strongly induced by submucosal BCG or MTBVAC vaccination than by standard intradermal vaccination (146). A variety of immunostimulatory adjuvants (e.g., bacterial toxins, TLR ligands, and cytokines) and nanoparticle adjuvants (e.g., virus-like particles, liposomes, and protoplasts) have been used in mucosal vaccines to enhance the immune responses (147).

## 8 Conclusion

Vaccines are powerful weapons for people to prevent and treatment many diseases. The sudden outbreak of the COVID-19 has pushed the development of vaccinology to a climax, and also provided valuable guidance for the development of TB vaccines. The BCG vaccine is undoubtedly one of the most potent weapons that humankind has acquired in the struggle against TB, but its limited protective effect is not sufficient to win the war. Based on the existing WHO-recommended immunization strategy for TB vaccines, protein subunit TB vaccines for specific populations (BCG-immunized, LTBI, and HIV-infected, etc.) have great potential for development and utilization. By far, multiple protein subunit TB vaccines have entered clinical or preclinical trials and have broken the barrier that BCG can only be used for pre-infection prevention. And even some vaccines have shown surprising protection in post-exposure prophylaxis in people with LTBI and in the treatment of people with active TB infection. Rapidly evolved bioinformatics and structural informatics technologies represent a large reservoir to filter out plentiful numbers of *Mtb-*protective antigens. Training immunity has been proposed in recent years and has received extensive attention in the field of TB. Trained immune cells are able to produce a rapid and effective protective response against *Mtb* attacks. Therefore, the activation of trained immunity should be considered in the development of vaccines and adjuvants. With the participation of various novel adjuvants, as well as the continuous optimization of animal models and vaccination strategies, effective protein subunit vaccines can be expected in the future to help achieve the grand goal of TB eradication.

## Author contributions

XF, and ZH contributed to the conception and revised of this manuscript. ZY and JX drafted and revised the manuscript. All authors contributed to the article and approved the submitted version.
